# Heat Shock Proteins and Inflammasomes

**DOI:** 10.3390/ijms20184508

**Published:** 2019-09-12

**Authors:** Pierre Martine, Cédric Rébé

**Affiliations:** 1 INSERM LNC-UMR1231, University of Bourgogne Franche-Comté, 21000 Dijon, France; piemartine@yahoo.fr; 2 Platform of Transfer in Cancer Biology, Centre Georges François Leclerc, 21000 Dijon, France

**Keywords:** heat shock proteins, inflammasomes, caspase-1, IL-1β

## Abstract

Heat shock proteins (HSP) regulate inflammation in many physiological contexts. However, inflammation is a broad process, involving numerous cytokines produced by different molecular pathways with multiple functions. In this review, we focused on the particular role of HSP on the inflammasomes intracellular platforms activated by danger signals and that enable activation of inflammatory caspases, mainly caspase-1, leading to the production of the pro-inflammatory cytokine IL-1β. Interestingly, some members of the HSP family favor inflammasomes activation whereas others inhibit it, suggesting that HSP modulators for therapeutic purposes, must be carefully chosen.

## 1. Introduction

### 1.1. HSP

Heat shock proteins (HSP) are molecular chaperones. Their primary role is to ensure the correct tri-dimensional conformation of proteins during their synthesis or upon cellular stress, and to prevent aggregation of wrongly folded proteins. Moreover, HSP are also involved in the elimination of incorrectly folded proteins, if repair is impossible. HSP are divided into six families depending on their molecular masses: large HSP or HSP100, HSP90, HSP70, HSP60, HSP40 and small HSP or sHSP.

#### 1.1.1. HSP100

The HSP100 family is a group of molecular chaperones able to solubilize almost all aggregated proteins in severe stress conditions. They are not required in normal physiological conditions and are synthesized following intense heat or other severe stresses [1]. ClpA was the first protein in the Hsp100 family to be discovered, as an important component of ATP-dependent protease.

By cooperating with other molecular chaperones such as HSP70, the HSP100 family is also involved in protein degradation, if repair is not possible [1].

#### 1.1.2. HSP90

HSP90 is a highly abundant molecular chaperone with diverse biological roles. Like other stress proteins, HSP90 is able to interact with non-native peptides and prevent their aggregation. HSP90 is functionally more specialized than other chaperones. This protein seems to act mainly at the late stages of substrate folding. For example, steroid hormone receptors must bind to HSP90 to be able to interact effectively with their ligand. The bacterial form of HSP90 appears to act alone and is not crucial for viability, but eukaryotic forms and their many cofactors are essential. It plays an important role in maturation of signaling proteins, in development and cellular division. Its substrates include steroid hormones receptors, kinases receptors and oncogenic proteins receptors [2].

HSP90 forms a dimer of elongate subunits, each subunit comprising three domains that are linked by flexible regions. HSP90 dimerizes stably, due to its C-terminal domains and also transiently through its N-terminal ATPase domain, when ATP binds to HSP90 [3]. Specific inhibitors of HSP90 ATPase domain have marked effects in development and cancer. Although the nucleotide state of HSP90 is weakly coupled to its conformational change, many HSP90 partners act on different stages of its functional cycle. HSP90 action is modulated by co-chaperones and client proteins (the term used for “substrates” in the HSP90 system). In addition, phosphorylation, acetylation, and other post-translational modifications affect its functional state [2].

The protein gp96 is the paralog of HSP90 in the endoplasmic reticulum and is involved in the maturation of Toll-like receptors [4].

#### 1.1.3. HSP70

HSP70 family members have three domains: an N-terminal ATPase domain, a centrally located substrate binding domain, and a C-terminal “Lid” domain, responsible for HSP70 substrate affinity. Their activity depends on dynamic interactions between ATPase and substrate-binding domains and also on the interactions between these domains and other co-chaperones, such as HSP40-like proteins and certain nucleotide exchange factors (NEFs), which stimulate ADP release and nucleotide exchange after ATP hydrolysis [5].

Transient binding of a polypeptide chain to HSP70 may prevent misfolding and aggregation and may also maintain the substrate in an unfolded state for translocation to another cellular compartment. The HSP70 system is an important component of the translocation system on both sides of organelle membranes. HSP70 intervenes both to deliver its protein-substrate to translocases that transport it across organelle membranes and to capture the translocated polypeptide [5].

Concerning its role as a chaperone, it appears that HSP70-related polypeptides resume their three-dimensional conformation automatically once released from HSP70. Thus, HSP70′s role seems to be to stabilize proteins in their unfolded form until they reach their target cell compartment, where they naturally acquire their final form [6].

In addition to its role as a chaperone, HSP70 has additional specific cellular functions. For example, when associated to auxilin (which is also a co-chaperone), it disassembles the clathrin mantle of membrane vesicles after completion of clathrin-mediated endocytosis [7]. HSP70 also cooperates with HSP100 ATPases to disaggregate large protein aggregates [8].

#### 1.1.4. HSP60

HSP60 family members are also called chaperonins. Chaperonins encapsulate non-native proteins in an ATP-dependent manner. In bacteria, the most common chaperonin is the GroEL protein, which associates with the GroES co-chaperone to form a molecular complex called GroE, composed of 14 subunits GroEL and a subunit GroES. In eukaryotes, this family is represented by HSP60 and HSP10 proteins, present in mitochondria with a function similar to their bacterial counterparts. GroEL encapsulates a non-native protein with support of a GroES co-chaperone in the presence of ATP. This mechanism allows for the correct folding of proteins [9].

#### 1.1.5. HSP40

HSP40 family members function in interaction with HSP70 family members, primarily by stimulation of their ATPase activity and by stabilization of their interaction with substrate proteins. HSP40 proteins all contain the J domain, which is responsible for their interaction with HSP70 [10].

#### 1.1.6. sHSP

sHSP chaperones are the most widespread but the lowest conserved HSP across species. sHSP show high heterogeneity in both sequence and size [11]. Their common trait is the conservation of an α-crystallin domain, in reference to the most prominent member of this family, the α-crystallin protein [12]. sHSP typically form large dynamic oligomers, often consisting of 24 subunits [13]. sHSP are chaperone proteins that act independently of ATP and interact with a large number of partially folded target proteins to prevent their aggregation after stress-induced conformational loss [14]. However, alone they are unable to correct this inaccurate conformation. According to current theory sHSP serve as a “storage repository” for non-native proteins, which can be folded in the presence of other chaperones such as HSP70 and HSP100 [15]. It seems that sHSP are not only able to form soluble complexes with their client proteins but, can also be sequestered in protein aggregates, in particular when protein denaturation is massive in the cell. This avoids formation of larger aggregates and facilitates the action of other chaperone proteins [16,17,18].

### 1.2. Inflammasomes

The connection between stimulus detection and inflammatory response consists of molecular complexes called inflammasomes. These intracellular complexes are all composed of a receptor and an adapter which allow recruitment and activation of pro-inflammatory caspases (cysteine proteases) as well as the maturation and secretion of pro-inflammatory interleukins such as IL-1β or IL-18 [19].

The receptors, called Nod (Nucleotide-binding oligomerization domain-containing protein 1)-like receptors or NLR, are encoded in humans by 23 different genes and recognize a wide variety of stimuli referred to as Pathogen-Associated Molecular Patterns (PAMPs). The NLR family is characterized by the presence of several specific domains. All of these proteins have a central NACHT (NAIP (neuronal apoptosis inhibitor protein), C2TA (MHC class 2 transcription activator), HET-E (incompatibility locus protein from Podospora anserina) and TP1 (telomerase-associated protein) domain responsible for complex activation, via ATP-dependent oligomerization. This domain is generally flanked at the C-terminal by a leucine-rich domain (LRR) involved in ligand detection and in complex self-regulation. N-terminal is either a caspase recruitment domain (CARD) or a pyrin domain (PYD) involved in protein-protein interactions for signal transduction. Thus the activated receptors can recruit either pro-caspases (via the CARD) or an adapter protein (via the PYD) which in turn, will recruit a pro-caspase. The NLR receptors are divided in four families according to N-terminal domain composition [20].

#### 1.2.1. NLRA

The NLRA subfamily is characterized by the presence of an acidic transactivation domain and is composed of only one member, the CIITA (Class II TransActivator) protein. CIITA was the first NLR described and is known for its role in the transcriptional regulation of MHC (Major Histocompatibility Complex) type II genes [21].

#### 1.2.2. NLRB

The subfamily NLRB is characterized by the presence of a N-terminal BIR (Baculovirus Inhibitor Repeat) domain. In humans, it contains only one member, the protein inhibitory neuronal apoptosis (NAIP), while 7 members are found in mice. NAIP detects bacterial flagellin or bacterial components of secretion system III (SST3), which leads to inflammasome formation. In mice, this role is shared between two types of NAIP proteins, NAIP5 and NAIP6, which detect flagellin, while NAIP2 detects SST3 proteins. NLRB receptors appear to associate to other NLRs to perform their function: in humans, NAIP associates with NLRC4 (or IPAF-Ice Protease-Activating Factor) to detect flagellin, while NAIP5, in mice also associates with NLRC4 to detect flagellin and NAIP2 partners with NLRC4 to detect SST3 proteins [22,23,24].

#### 1.2.3. NLRC

The NLRC subfamily is characterized by the presence of a CARD caspase recruitment domain and/or the absence of a PYD domain.

Nod1 (NLRC1) and Nod2 (NLRC2) are two cytoplasmic proteins activated by the recognition of specific motifs present in the bacterial peptidoglycan. Nod1 is found in the vast majority of cells, whereas Nod2 has been found in macrophages, dendritic cells, Paneth cells, keratinocytes, intestinal epithelium, lungs, oral cavity and osteoblasts [25]. Once activated, Nod1 and Nod2 will become oligomerized via their NACHT domain, thus creating a protein platform which allows signaling proteins activation and recruitment of RIP1 protein via its CARD. Nod1 and Nod2 activation, allows NF-κB (Nuclear Factor-kappa B) and stress kinases pathways regulation [26].

NLRC3 is expressed in T cells and NK cells (Natural Killer) and has an anti-inflammatory role. NLRC3 inhibits TLR-mediated NF-κB activation by binding to the TRAF6 (TNF Receptor Associated Factor 6) adapter protein. Binding leads to inhibition of TRAF6 K-63 ubiquitination, resulting in loss of function and inhibition of NF-κB pathway [27].

As previously described, NLRC4 associates with NAIPs to detect bacterial compounds and form a functional inflammasome. Its activation leads to IL-1β and IL-18 production [28].

NLRC5 is expressed in macrophages, dendritic cells and B and T lymphocytes. This protein is involved in the regulation of MHC type 1 expression via direct interaction with the gene promoter. NLRC5 is induced by bacterial compounds such as poly (I:C) and CpG or by type I and II interferons [29].

NLRX1 is a mitochondrial receptor detecting single and double-stranded RNA. Its activation induces ROS (Reactive Oxygen Species) production. NLRX1 has anti-inflammatory activity via inhibition of the MAVS pathway (Mitochondrial AntiViral-Signaling Protein), and subsequent NF-κB pathway [30].

#### 1.2.4. NLRP

The NLRP subfamily comprises 14 members and is characterized by the presence of a N-terminal PYD domain. NLRP fall into different categories: The first category includes NLRPs unable to form a functional inflammasome or whose inflammasome has not yet been observed. It consists of NLRP4, 5, 8, 9, 10, 11, 13 and 14. The second category consists of NLRP1, 2, 3, 6, 7 and 12, which can form a functional inflammasome composed of the NLRP receptor, the ASC (Apoptosis associated Speck-like protein containing a CARD domain) adapter protein and pro-caspase-1 [31].

Members of the first category of NLRP, the ones that do not form an inflammasome, are mostly found in oocytes, embryos and testes where they play a major role in the fertility or embryo development [32,33,34]. Some of these NLRP also have other functions. NLRP4 is able to bind to the Beclin1 protein via the interaction of their NACHT domain. Beclin1 is a molecule important in autophagy, and this interaction causes the inhibition of this phenomenon [35]. NLRP4 is also able to negatively regulate the production of type 1 interferon by associating with DTX4 (Deltex E3 ubiquitin ligase 4). This association leads the proteasome addressing of TBK1 (TANK-Binding Kinase 1), an essential molecule in the production of type 1 interferons. NLRP10 is the only NLR that does not have a LRR domain, which has led to the postulate that it acted as a negative regulator of the other NLRs [36]. NLRP10 is also involved in Th1 and Th17 response to *candida albicans* infections [37]. NLRP10 also plays a role in the inflammatory response associated with Nod1, suggesting a cooperation between this two proteins [38].

NLRP1 is found ubiquitously in all cell types. It is activated by muramyldipeptide, a peptidoglycan component of bacteria wall, by the lethal toxin of anthrax (LeTx) and by a decrease of intracellular ATP concentration. In rodents, NLRP1 activation has been observed following *Toxoplasma gondii* infection. Its activation leads to ASC and pro-caspase-1 recruitment and to the secretion of IL-1β and IL-18 [39].

NLRP2 forms a functional inflammasome in human astrocytes by interacting with the P2X7 membrane receptor and Pannexin 1 following cell treatment with extracellular ATP [40]. NLRP2 also plays an essential role in mouse embryonic development since its depletion causes developmental arrest [41].

NLRP3 is the most studied NLR due to its involvement in several pathologies such as Alzheimer’s disease, atherosclerosis, cancer or allergy. Prior to its activation, expression of NLRP3 protein must be up-regulated through NF-κB activation, a mechanism called “priming”. NLRP3 is activated by a wide variety of stimuli and by three non-exclusive pathways, with a possible crosstalk. The first pathway involves binding of extra-cellular ATP to its receptor P2X7, leading to intracellular K^+^ efflux. The second pathway involves crystalline structures phagocytosis and subsequent lysosome damage. Lysosomal content, especially cathepsin B, will then activate NLRP3 through a direct interaction. The third pathway involves an increase in ROS synthesis leading to NLRP3 activation, recruitment of ASC and pro-caspase-1 and IL-1β and IL-18 secretion [42].

NLRP6 is highly expressed in intestinal tissues and inflammasome formation protects the body against inflammatory colitis and colon cancer [43]. Regardless NLRP6 inflammasome formation, NLRP6 is also involved in NF-κB signaling and in the regulation of mucus and antimicrobial peptides secretion [44].

NLRP7 is predominantly present in testis but is also able to form a functional inflammasome in human macrophages. Its activation by microbial lipopeptides leads to IL-1β and IL-18 secretion [45].

NLRP12 is expressed in dendritic cells and neutrophils and is linked to atopic dermatitis and recurrent hereditary fevers in humans. NLRP12 is responsible for the production of IL-1β and IL-18 after infection with certain pathogens such as *Yersinia pestis* [46]. In the absence of NLRP12, dendritic cells and neutrophils express CCR7 and CXCR4, but are unable to respond to their ligands, CCL19, CCL21 and CXCL12, which alters dendritic cells migration to lymph nodes [47].

## 2. Positive Effects of HSP on Inflammasomes Activation

### 2.1. HSP60

In the microglia, HSP60 is required for phosphorylation and nuclear localization of NF-κB after stimulation by the pro-inflammatory cytokine IL-1β. HSP60 knockdown leads to the inhibition of the NF-κB p65 subunit phosphorylation and in consequence to the inhibition of the nuclear translocation of NF-κB, suggesting that HSP60 is necessary for p65 phosphorylation. This NF-κB pathway activation leads to the overexpression of both pro-IL-1β and NLRP3, which corresponds to the “priming” step of the NLRP3 inflammasome activation. HSP60 also induces mitochondrial damages as shown by a decrease in mitochondrial membrane potential following HSP60 overexpression and IL-1β treatment. This entails an increase in the production of ROS, leading to oxidative stress. This oxidative stress activates the NLRP3 inflammasome [48].

### 2.2. HSP70

Extracellular HSP70 binds to the plasma membrane of human monocytes. This leads to an increased pro-IL-1β, TNFα and IL-6 expression in a CD14 and NF-κB-dependent manner, suggesting that extracellular HSP70 can prime immune cells for further inflammasome activation. More specifically, treatment of monocytes with extracellular HSP70 and its interaction with the receptor CD14 results in the phosphorylation of I-κBα on its serine 32 (Ser32), leading to its degradation by the proteasome and the release of functional NF-κB. NF-κB will then translocate to the nucleus and cause the overexpression of several pro-inflammatory molecules such as pro-IL-1β. However, there is currently no further data on whether this increase of pro-IL-1β production will translate into an increased IL-1β secretion following inflammasomes activation [49].

### 2.3. HSP90

HSP90, in conjunction with its partner SGT1 (Suppressor of G-Two allele of skp1), is able to interact with several NLRs via their LRR and NACHT domains. Such interactions are observed in vitro with NLRP2, NLRP3, NLRP4, NLRP12, Nod1, Nod2 and IPAF.

In the context of NLRP3 activation by monosodium urate (MSU) crystals and bacterial peptidoglycan, inhibition of either SGT1 via siRNA or HSP90 using geldanamycin, which competes with ATP binding to HSP90, results in a decrease of NLRP3-mediated inflammation. This shows an essential role of both SGT1 and HSP90 in NLRP3-containing inflammasomes function [50,51]. This result is due to NLRP3 and pro-IL-1β stabilization by fixation of the SGT1/HSP90 complex on the LRR domain of NLRP3, leading to their protection from degradation by autophagy [50,52]. Inhibition of HSP90 also leads to a decrease in Nod2 and IPAF activity, although the mechanism behind this regulation is not known.

In contrast to its effects on NLRP3, Nod2 and IPAF-mediated inflammation, HSP90 was shown to have no effect on NLRP1-mediated inflammation, even though a heat shock leading to its overexpression inhibits NLRP1 inflammasome activation after stimulation with anthrax lethal toxin [53].

In the context of subarachnoid hemorrhage in mice, inhibition of HSP90 by 17-AAG (17-allylamino-17-demethoxygeldanamycin) was shown to inhibit NLRP3 inflammasome activation, whereas the transfection of recombinant HSP90 (rHSP90) increases NLRP3 activation and abolishes the effect of 17-AAG. This effect of HSP90 on the activation of NLRP3 involves the ATP receptor P2X7, as the inhibition of this receptor abolishes the increased NLRP3 activation after transfection with rHSP90 [54]. Another HSP90 inhibitor, AT-533 inhibits herpes simplex virus (HSV-1)-mediated HSP90-NLRP3 interaction and IL-1β production and also inhibits HSV-1 induced NF-κB signaling, leading to a decrease in the synthesis of pro-IL-1β [55].

### 2.4. Gp96

Extracellular heat shock protein gp96 is able to activate NLRP3 inflammasome in antigen presenting cells. Firstly, treatment of these cells with gp96 leads to an increased pro-IL-1β and NLRP3 protein expression. This increase is the result of the interaction between gp96 and the receptor CD91, which will subsequently activate both the NF-κB and MAPK (Mitogen-Activated Protein Kinase) pathways. Inhibition of either NF-κB with cardamonin or MAPK with SB203580 abolishes the NLRP3 inflammasome activation by extracellular gp96. This indicates an involvement of gp96 in the activation of the “priming” step of the NLRP3 inflammasome [56].

Extracellular gp96 is also able to directly activate NLRP3 inflammasome, leading to a release of IL-1β by macrophages. The exact mechanism behind this effect is unknown, but the activation of NLRP3 by gp96 and the subsequent IL-1β release require K^+^ efflux. Interestingly, this activation is not dependent on P2X7 receptor, suggesting a different pathway from the ATP-mediated K^+^ efflux. This is reinforced by the notable differences in NLRP3 inflammasome activation kinetics between gp96 and other activators. Gp96 slower activation kinetics could suggest the need of a constant cell exposure to gp96 or that gp96 activates NLRP3 in an indirect manner, requiring other endogenous signals [56].

## 3. Negative Effects of HSP on Inflammasomes Activation

### 3.1. HSP27

In the context of atherosclerosis, extracellular HSP27 has been shown to inhibit the secretion of IL-1β [57]. Extracellular HSP27 inhibits acLDL uptake by competing for the fixation of acLDL to the Scavenger Receptor A (SR-A). This effect is abolished when the macrophages are treated with the SR-A competitive ligand fucoidan and when using macrophages deficient for SR-A. This competition between HSP27 prevents acLDL uptake by macrophages and thus inhibits lysosomal rupture and subsequent NLRP3 inflammasome activation by cholesterol crystals [58].

Intracellular HSP27 is also able to inhibit inflammatory responses through NF-κB downmodulation pathway in macrophages, by inhibiting the degradation of the inhibitor IκBα following NF-κB activating signals [59,60,61], suggesting a possible inhibition of the priming step.

Classical monocytes have been shown to be more prone to produce IL-1β than non-classical monocytes under BzATP treatment. This seems to be related to HSP27 overexpression in non-classical monocytes, as siRNA targeting this HSP increases IL-1β secretion, enhancing HSP27 inhibitory effect on inflammasomes [62].

### 3.2. HSP70

**Figure 1:**
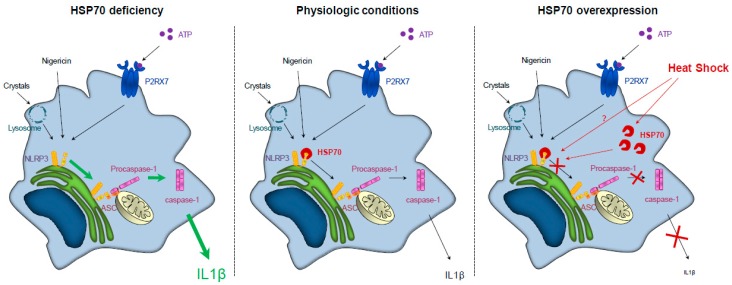
HSP70 (Heat Shock Protein 70) expression regulates NLRP3 (NOD-leucine rich repeat and pyrin containing protein 3) inflammasome activation. Left panel: HSP70 deficiency leads to an overactivation of NLRP3 inflammasome and caspase-1 and to an increased secretion of IL (interleukin)-1β. Middle panel: In physiologic conditions, NLRP3 inflammasome activation and IL-1β secretion are regulated. Right panel: HSP70 overexpression (artificially or through a heat shock) leads to the continued interaction between HSP70 and NLRP3, preventing the interaction between NLRP3 and ASC (Apoptosis associated Speck-like protein containing a CARD domain) and the subsequent caspase-1 activation and IL-1β secretion.

HSP70 has been shown to inhibit NLRP3 inflammasome activation. Inhibition of HSP70 in vivo or in vitro worsens MSU or alum-induced NLRP3-dependent peritonitis in mice. HSP70 deficiency also enhances caspase-1 activation and IL-1β production by murine Bone Marrow-Derived Macrophages (BMDMs) treated with NLRP3 activators in vitro. These phenomena are associated with an increase in the number and size of ASC/NLRP3 specks, denoting an increase in the activation of the NLRP3 inflammasome and its complex formation. In contrast, HSP70 overexpression in BMDMs decreases NLRP3/ASC interaction, caspase-1 activation and IL-1β production upon treatment with NLRP3 activators in vitro. This inhibitory effect could be explained by an interaction between HSP70 and NLRP3 (Figure 1). Further studies are required to know how this interaction prevents the adaptor ASC to interact with NLRP3 and to recruit the pro-caspase-1. The two main hypotheses are either a conformational modification of NLRP3 by HSP70 or a competition between HSP70 and ASC on the PYD of NLRP3. Interestingly, the HSP70/NLRP3 interaction is lost under physiological NLRP3 activation. A heat shock, used as a way to induce HSP70 expression, also inhibits the NLRP3 inflammasome activation in vitro. Moreover, in vivo hyperthermia leads to HSP70 overexpression and inhibits MSU or alum-induced NLRP3-dependent peritonitis features in mice, as shown by a decrease of IL-1β secretion by peritoneal macrophages and a decrease of neutrophils influx [63].

HSP70 chaperone function can also be responsible for its inhibitory effect. The ability to dissolve protein aggregates that would otherwise activate the NLRP3 inflammasome might be an explanation for this observation. An illustrating example is its ability, in association with HSP40 and members of the HSP100 family, to dissolve amyloid filaments aggregates [64].

## 4. Impact of HSP Polymorphisms on Inflammatory Diseases Development

IL-1β, the final cytokine produced after inflammasome activation is implicated in many inflammatory diseases [65]. NLRP3 participates in the inflammatory component of several diseases such as multiple sclerosis [66,67], gouty arthritis [68], atherosclerosis [58], Alzheimer’s and Parkinson’s diseases [69,70], type 2 diabetes [71], or cancer [72]. Some inflammatory diseases are induced by polymorphisms or mutations in genes coding for constituents of inflammasomes. Thus, NLRP3 mutations lead to cryopyrin-associated periodic syndromes (CAPS), such as familial cold autoinflammatory syndrome, Muckle–Wells Syndrome and neonatal onset multi-systemic inflammatory disease/chronic infantile neurological cutaneous articular syndrome [73].

Because HSP can modulate inflammasome activation, one can speculate that HSP mutations or polymorphisms will influence inflammasome activation and IL-1β production and will predispose patients to inflammatory diseases. In humans, several polymorphisms on genes coding for HSP70 have been reported. HSP70.2 + 1267 G/A polymorphism is correlated with a lower expression of HSP70 protein in peripheral blood mononuclear cells from multiple sclerosis patients [74]. Moreover, this polymorphism is associated with a higher risk of developing inflammatory diseases, such as Crohn’s disease, multiple sclerosis, pancreatitis or systemic erythematous lupus [74,75,76,77,78]. An association between rheumatoid arthritis and polymorphism in the promoter of HSP70 gene was described. A similar association between the coding region of HSP70-Hom, a variant of HSP70, and rheumatoid arthritis is controversial [79].

Inflammatory diseases involved many inflammatory pathways (not only inflammasome over activation). Further work is needed to understand how described HSP polymorphisms can influence the development of such pathologies and to search for new HSP polymorphisms that can be related to inflammasome deregulation and inflammasome-dependent disease development.

## 5. Modulation of HSP Expression or Activity in Inflammatory Diseases

While most HSP favor inflammasomes activation, only HSP27 and intracellular HSP70 are able to inhibit it (Table 1). HSP70 has an ambivalent role depending on its localization. When extracellular, it is an inducer of pro-IL-1β expression [49], while when intracellular it is an inhibitor of NLRP3 inflammasome activation [63]. These effects render difficult HSP70 expression modulation in the context of inflammasome activation inhibition. However, we have used heat shock to increase HSP70 expression and were able to inhibit NLRP3 activation and IL-1β production [63]. Heat shock can also be used to induce HSP27 expression. However we have noticed that in monocytic cells a heat shock of 42 °C for 1 h was able to increase HSP70 expression after 2 h of resting, whereas HSP27 expression is observed 24 h after resting. Therefore, efforts must be taken to overexpress the desired HSP [63].

A balance between HSP90 and HSP70 on NLRP3 inflammasome activation can be proposed. HSP90 inhibition by geldanamycin and overexpression of HSP70 both inhibit NLRP3 inflammasome activation [50]. However it has been shown that geldanamycin can increase HSP70 expression [80] and that its effects can also be mediated by HSP70 induction and not HSP90 inhibition [81], thus suggesting that geldanamycin can act on both HSP. Concerning other HSP, inhibitors can be used to treat inflammatory diseases. An important work has already been done to develop HSP inhibitors, which may be attractive to therapeutic targets in many diseases such as cancer [82], hematopoietic malignancies [83], or Alzheimer’s disease [84].

## 6. Conclusions

As described in this review, several HSP have a strong impact, positive or negative, on inflammasome activation. These observations demonstrate an interest in adjusting HSP expression or activity to modulate inflammation. However, in the context of inflammatory disorders treatment, the ambivalent effect of these chaperone proteins encourages us to carefully pinpoint the target HSP and to consider not only inhibition of HSP but also their induction/activation, depending on the physiopathologic context.

## Figures and Tables

**Table 1:** Effects of HSP on the NLRP3 inflammasome.

HSP	Inflammasome Activation	Inflammasome Inhibition	Mechanism
Ext HSP27		+	Competition with acLDL
Int HSP27		+	Unknown
HSP60	+		NF-κB activation/ROS
Intra HSP70		+	Interaction with NLRP3
Ext HSP70	+		NF-κB activation
HSP90	+		Stabilization of the complex with SGT1 and interaction with NLRP3
Gp96	+		NF-κB activation or K+ efflux
